# Microtubules Stabilization by Mutant Spastin Affects ER Morphology and Ca^2+^ Handling

**DOI:** 10.3389/fphys.2019.01544

**Published:** 2019-12-20

**Authors:** Nicola Vajente, Rosa Norante, Nelly Redolfi, Andrea Daga, Paola Pizzo, Diana Pendin

**Affiliations:** ^1^Department of Biomedical Sciences, University of Padua, Padua, Italy; ^2^Laboratory of Molecular Biology, Scientific Institute IRCCS E. Medea, Lecco, Italy; ^3^Neuroscience Institute—Italian National Research Council (CNR), Padua, Italy

**Keywords:** spastin, drosophila, microtubules, endoplasmic reticulum, calcium, SOCE, calcium imaging

## Abstract

The endoplasmic reticulum (ER) extends as a network of interconnected tubules and sheet-like structures in eukaryotic cells. ER tubules dynamically change their morphology and position within the cells in response to physiological stimuli and these network rearrangements depend on the microtubule (MT) cytoskeleton. Store-operated calcium entry (SOCE) relies on the repositioning of ER tubules to form specific ER-plasma membrane junctions. Indeed, the tips of polymerizing MTs are supposed to provide the anchor for ER tubules to move toward the plasma membrane, however the precise role of the cytoskeleton during SOCE has not been conclusively clarified. Here we exploit an *in vivo* approach involving the manipulation of MT dynamics in *Drosophila melanogaster* by neuronal expression of a dominant-negative variant of the MT-severing protein spastin to induce MT hyper-stabilization. We show that MT stabilization alters ER morphology, favoring an enrichment in ER sheets at the expense of tubules. Stabilizing MTs has a negative impact on the process of SOCE and results in a reduced ER Ca^2+^ content, affecting the flight ability of the flies. Restoring proper MT organization by administering the MT-destabilizing drug vinblastine, chronically or acutely, rescues ER morphology, SOCE and flight ability, indicating that MT dynamics impairment is responsible for all the phenotypes observed.

## Introduction

The endoplasmic reticulum (ER) coordinates a variety of cellular processes, such as synthesis, modification, quality control and transport of proteins, as well as lipid metabolism and Ca^2+^ homeostasis. It extends as a single membrane-bound entity composed of interconnecting sheets and tubules spreading all over the cell. Although the ER can form a reticular network independently of cytoskeletal structures (Dreier and Rapoport, 2000), in mammalian cells its distribution and sheet/tubule balance are influenced by microtubules (MTs) (Terasaki et al., 1986; Dabora and Sheetz, 1988; Lee and Chen, 1988; Waterman-Storer and Salmon, 1998; Lu et al., 2009; Joensuu et al., 2014).

MTs are composed of tubulin polymers and constitute essential components of the cytoskeleton. In neurons, they are critical in order to support long-range motor-driven cargo transport within neuronal processes and play fundamental roles in polarity, axon differentiation and growth (Conde and Cáceres, 2009; Kapitein and Hoogenraad, 2015). Although a part of the neuronal MTs is considered stable, a fraction retains high levels of dynamics, as demonstrated by their frequent and continuous growth and shortening (Desai and Mitchison, 1997; Nogales, 2001; Burbank and Mitchison, 2006). This dynamic instability is central to MT biological functions, allowing their rapid reorganization at need (Kirschner and Mitchison, 1986). The organization of MTs in neurons is tightly regulated by assembly-promoting factors, stabilizing and destabilizing factors and severing proteins. Dysfunctional MTs, due to mutations in genes that encode tubulin or MT-associated proteins, have been linked to a range of neuronal diseases, such as motor neuropathies, Hereditary Spastic Paraplegias (HSPs), Charcot–Marie–Tooth disease.

Two major types of MT-dependent ER movement have been described: sliding, the motor-based transfer along stable, pre-existing MTs; and movement mediated by the tip attachment complex (TAC), by which a plus end-attached ER tubule extends together with a MT growing end (Waterman-Storer and Salmon, 1998; Friedman et al., 2010). TAC has been proposed to have a role in one of the major functions of ER, i.e., intracellular Ca^2+^ handling. Indeed, the ER lumen contains a 10,000-fold higher Ca^2+^ concentration than that of the bulk cytosol, working as the primary intracellular Ca^2+^ store, releasing Ca^2+^ in the cytosol upon different cellular stimulations (Zampese and Pizzo, 2012; Pendin et al., 2017). The main source of Ca^2+^ for ER refilling is the extracellular space, and the plasma membrane (PM) is contacted by ER tubules in a process called store-operated Ca^2+^ entry (SOCE) (Várnai et al., 2009), which serves to generate a sustained cytosolic Ca^2+^ elevation and refill the depleted ER Ca^2+^ store.

The molecular players involved in SOCE include the pore-forming subunit of the Ca^2+^-release activated Ca^2+^ channel encoded by the *Orai* gene (Feske et al., 2006; Prakriya et al., 2006; Vig et al., 2006a,b; Yeromin et al., 2006; Zhang et al., 2006) and the ER-resident protein STIM (stromal interaction molecule) (Liou et al., 2005; Zhang et al., 2005), that serves as a luminal Ca^2+^ sensor (Grigoriev et al., 2008; Friedman et al., 2010; Soboloff et al., 2012). It has been demonstrated that after Ca^2+^ store depletion, STIM oligomerizes and redistributes to predetermined foci in the peripheral ER (Luik et al., 2008). STIM binds the MT plus-end binding protein EB1, which facilitates TAC-dependent STIM translocation toward the PM (Liou et al., 2007; Honnappa et al., 2009; Chen et al., 2013, 2019; Tsai et al., 2014). At the ER–PM junctions, STIM interacts with Orai channels to promote influx of extracellular Ca^2+^ into the ER (Liou et al., 2007; Grigoriev et al., 2008; Galán et al., 2011). In this STIM redistribution process, the physical movement of ER is required for ER tubules to reach out to the PM and form new ER-PM junctions (Wu et al., 2006; Carrasco and Meyer, 2011). The precise role of TAC-based ER movement in this reorganization, however, is controversial and variable among cell types (Redondo et al., 2006; Smyth et al., 2007; Grigoriev et al., 2008; Galán et al., 2011). One model proposes that TAC-mediated ER movement is required prior to SOCE activation to appropriately locate STIM on ER membrane, while ER Ca^2+^ depletion causes MT-independent STIM translocation to the PM (Smyth et al., 2007). Although the molecular details of this process are unclear, local cytoskeleton reorganization is supposed to play a major role (Gurel et al., 2014).

Spastin is an ATPase with MT-severing activity (Hazan et al., 1999; Errico et al., 2002; Roll-Mecak and McNally, 2010; Sharp and Ross, 2012; Sandate et al., 2019). Mutations in the *spastin* gene cause over 50% of cases of pure autosomal dominant HSPs, a group of neurodegenerative disorders characterized by lower-limb spasticity and weakness (Fink, 2013); primarily due to degeneration of the descending axons of cortico-spinal neurons. Fly models for spastin-dependent HSP have been created both by inactivating protein function (Sherwood et al., 2004; Trotta et al., 2004) or by expressing a pathogenic mutant version of fly spastin (Orso et al., 2005). Despite the extensive progress in the comprehension of spastin functions, the specific mechanisms by which its mutants lead to HSPs remain unclear. Spastin has been implicated in axonal transport (Errico et al., 2002; Yu et al., 2008; Kasher et al., 2009; Fassier et al., 2013), neuromuscular junctions (NMJ) morphology and function (Sherwood et al., 2004; Trotta et al., 2004) and axon guidance (Wood et al., 2006; Butler et al., 2010), suggesting that its role in maintaining neuronal heath is likely related to its MT severing activity.

Here we show that MT alteration due to the expression of spastin carrying the pathogenic mutation K467R reduces SOCE and decreases ER Ca^2+^ content in Drosophila neurons. ER morphology appears altered, as an increase in ER sheets is observed at the expense of tubules. Importantly, both morphological and functional ER defects are rescued when flies are exposed to the MT-destabilizing drug vinblastine, indicating that rescue of MT structure is sufficient to restore ER normal shape and function.

## Materials and Methods

### Drosophila Stocks and Crosses

The UAS-Dspastin-K467R and UAS-BiP-sf-GFP-ER fly lines used in this study were described previously (Orso et al., 2005; Summerville et al., 2016). The Gal4 strains used were: Elav-Gal4 (pan neuronal expression); D42-Gal4 (motor neurons restricted), obtained from Bloomington Drosophila Stock Center. To increase protein expression, all experimental crosses were performed at 28°C. Control genotypes included promoter-Gal4/+ individuals. Fly food was prepared using NUTRI-fly-IF mixture (Genesee Scientific), according to the manufacturer instructions. For chronic vinblastine treatment, NUTRI-fly-IF was additioned with 50 nM vinblastine.

### Electron Microscopy

Larval brains were fixed in 4% paraformaldehyde and 2% glutaraldehyde and embedded as previously described (Orso et al., 2009). Electron microscopy images were acquired from thin sections under a FEI Tecnai-12 electron microscope at the DeBio imaging Electron Microscopy Facility (University of Padova).

### Confocal Images of Larval Brains

Brains and ventral ganglia from third instar larvae expressing BiP-sf-GFP-ER alone or together with spastin^K467R^ were dissected in M1 medium (see below) containing 1 mM Ca^2+^, then motor neuron cell bodies were imaged on a Leica TCS SP5 II confocal microscope equipped with a HCX PL APO lambda blue 63x/1.40-0.60 Oil objective, using a 488 nm laser.

For the quantification of ER distribution along nerves, BiP-sf-GFP-ER fluorescence was measured in regions located near the ganglion, along the axon, and at the end of the larval body. Mean fluorescence was calculated using ImageJ software.

### Protein Extraction and Western Blotting

Proteins were extracted from 15 flies expressing BiP-sf-GFP-ER alone or together with spastin^K467R^ under the control of the motoneuron promoter D42-Gal4. GRS Full Sample Purification Kit (GRiSP, Lda.) was used according to the manufacturer's instructions. The protein pellet was solubilized in 80 μL of RIPA Buffer (50 mM Tris, 150 mM NaCl, 1% Nonidet P-40, 0.5% deoxycolic acid, 0.1% SDS, pH 7.5), supplemented with proteases and phosphatases inhibitors mixtures (Roche, 04693132001 and 04906837001) and 3 M urea. Insoluble particles were spun down at 10,000 g for 5 min. Proteins were separated by SDS-PAGE, transferred into nitrocellulose membranes (GE Healthcare, 10600001) and probed using the following antibodies: anti-GFP (Cell Signaling, 2956S), 1:1000; anti-ACT (beta-actin) (Sigma Aldrich, A2228), 1:2500. The intensity of the bands was analyzed using ImageJ software.

### Preparation of Larval Neurons

Larval neurons were dissociated as previously reported (Chakraborty and Hasan, 2018). Briefly, third instar larvae were collected in a Petri dish, rinsed once with double-distilled water, twice with 70% ethanol, then with M3 complete medium (Shields and Sang M3 Insect Medium, supplemented with 10% heat-inactivated FBS, 50 U/mL penicillin, 50 μg/mL streptomycin). Brains were dissected with sterilized forceps under a light microscope. Brains were washed twice with M3 complete medium, then transferred to an enzymatic solution (0.75 μg/μL collagenase A and 0.4 μg/μL dispase II in M3 complete medium) and incubated for 20 min at room temperature in agitation. During incubation, brains were mechanically dissociated by gentle pipetting. Cell lysates were centrifuged at 600 × g for 5 min in a table top centrifuge and washed twice with dissecting solution to remove any residual enzymes. The cell pellet was resuspended with 100 μL of M3 complete medium for each brain; 100 μL were plated for each coverslip, approximately corresponding to one brain. Coverslips were previously autoclaved and coated with a drop of 0.1 mg/mL of poly-L-lysine for 30 min at 37°C.

### Climbing Assay

Climbing assay was performed as previously described (Agrawal and Hasan, 2015) using a 2.5 cm diameter glass cylinder. A group of 20 seven-days-old flies of the indicated genotype were dropped in the cylinder and a gentle taps were given to convey the flies to the bottom of the cylinder. The number of flies that crossed a mark drawn 10 cm above the bottom of the tube in a 60 s time window was counted manually. Each batch of flies was tested three times. The number of climbing flies for each batch was calculated as the mean of the climbing flies in the three repetitions. The total number of climbing flies for each genotype was calculated as the sum of the means. An independent proportion analysis was used to determine statistical differences between populations.

### Flight Assay

The flight assay was adapted from a previously published protocol (Banerjee, 2004) using a 1 m long, Plexiglas cylinder (diameter 5 cm) connected with an ethanol filled chamber at the bottom. Groups of 20 flies of a selected genotype were dropped into the cylinder through the top entry. A fly was determined to be capable of flight if it manages to reach the cylinder wall. Flies that could not perform this task fell directly to the ethanol filled chamber. Flight assays were performed on day 7 post eclosion. Data represent the percentage of flies capable of flight, at least 100 flies per condition were tested. Independent proportion analysis was used to determine the differences between groups.

### Cytosolic Ca^2+^ Imaging

Neuronal cells were incubated with fura-2/AM (1 μM), pluronic F-127 (0.02%), and sulfinpyrazone (200 μM) for 20 min at room temperature (RT) in a M1 buffer (see below) and then in a fresh solution without the Ca^2+^ indicator for 20 min at RT. Fura-2–loaded cells were visualized with a 20x ultraviolet-permeable objective (CFI Sfluor 20x N.A. 0.75, Nikon) on an inverted microscope (Nikon Ti-E). Fluorescence illumination was achieved by 50–75W Lamp (USHIO UXLS50A) and alternating excitation wavelengths (340/380 nm) were obtained by a monochromator (Optoscan CAIRN-Research) controlled by NIS-ELEMENTS AR (Nikon) software. A neutral density filter, ND4 (Nikon, USA) and a FF-409-DiO3 Dichroic (Semrock) were used in the excitation pathway. The emitted fluorescence was collected using a 510/84 nm (Semrock) filter. Images were acquired every 1 s, with 100 ms exposure time at each wavelength, by a Zyla-CMOS 4.2-P (Andor, Oxford Instruments) controlled by the same software. During the experiment, cells plated on coverslips were mounted into an open-topped chamber and maintained in an extracellular-like medium containing the following:

(1) M1 (Na^+^-based) medium: 30 mM HEPES, 150 mM NaCl, 5 mM KCl, 1 mM MgCl2, 35 mM sucrose, 5 glucose, pH 7.2 with NaOH at RT;

(2) K^+^-based medium: 30 mM HEPES, 145 mM K-D-gluconate, 10 mM NaCl, 1 mM MgCl_2_, 35 mM sucrose, 5 mM glucose pH 7.2 with KOH at RT.

For store Ca^2+^ content evaluation, cells were firstly perfused with M1 containing 1 mM CaCl_2_; after addition of 500 μM EGTA, cells were stimulated by addition of ionomycin (10 μM) or cyclopiazonic acid (CPA, 50 μM). In the second case, for residual Ca^2+^ evaluation, cells were further stimulated with addition of ionomycin (10 μM). For SOCE activation experiments, cells were pre-treated with the irreversible SERCA inhibitor thapsigargin (100 nM) for 10 min in a Ca^2+^-free, EGTA (500 μM)-containing M1; cells were then perfused with the same medium without the SERCA inhibitor and challenged with CaCl_2_ (2 or 5 mM). Where indicated, M1 (Na^+^-based) medium was substituted with K^+^-based medium.

For acute vinblastine treatment, the drug (1 μM) was added in each solution and step of the experimental protocol, from fura-2/AM cell loading to cell stimulations. Neurons dissociated from larvae exposed to chronic vinblastine treatment, were similarly treated.

### Ca^2+^ Imaging Experiments Analysis

Off-line analysis of Ca^2+^ imaging experiments was performed using the NIS-Elements software. F_340_ and F_380_ images were subtracted of background signals and proper regions of interest (ROIs) were selected on each imaged cell. The ratio of the emitted fluorescence intensities (R = F_340_/F_380_) was calculated for each ROI, normalized to the value measured before stimulus addition, or at the Ca^2+^-free status, and averaged offline. Data were analyzed using Microsoft Excel and Graphpad Prism 8 to calculate areas under the curves (AUC).

### Statistical Analyses

Fura-2 traces represent average values of 100 to 1,000 cells collected in 3–10 independent experiments. Average values are expressed as mean ± standard error of the mean (*n* = number of cells, unless otherwise specified). Statistical analyses were performed using unpaired Student's t-test. Analyses of differences between fly populations were made using chi-square independent proportion analysis. Both tests were applied with a confidence interval of 95% (*p < 0.05, **p < 0.01, ***p < 0.001).

### Materials

Shields and Sang M3 Insect Medium, Dispase II, vinblastine, thapsigargin, EGTA and CaCl_2_ were purchased from Sigma-Aldrich. CPA, and ionomycin were purchased from Abcam, Collagenase A was purchased from Roche. Fura-2/AM was purchased from Thermo Fisher. All other materials were analytical or of the highest available grade.

## Results

### Neuronal Expression of Spastin^K467R^ Influences ER Morphology

To alter MT stability, we used a transgenic line for the expression of Drosophila spastin carrying the mutation K467R under the control of UAS promoter (UAS-Dspastin-K467R). The amino acid substitution, located in the AAA ATPase domain, corresponds to the pathogenic mutation K388R in the human spastin protein, known to produce a dominant-negative effect (Orso et al., 2005). Indeed, when spastin^K467R^ is expressed in a wild-type background, hyper-stabilization of MTs has been observed, similar to that produced by downregulation of spastin (Orso et al., 2005). We expressed spastin^K467R^ in the fly nervous system, using the pan-neuronal driver elav-Gal4. The birth rate of flies expressing spastin^K467R^ was partially reduced, compared to control flies (Supplementary Figure 1A). Moreover, we confirmed that these flies show a shorter lifespan and locomotor dysfunction (Supplementary Figures 1B,C), as previously reported (Orso et al., 2005).

**Figure 1:**
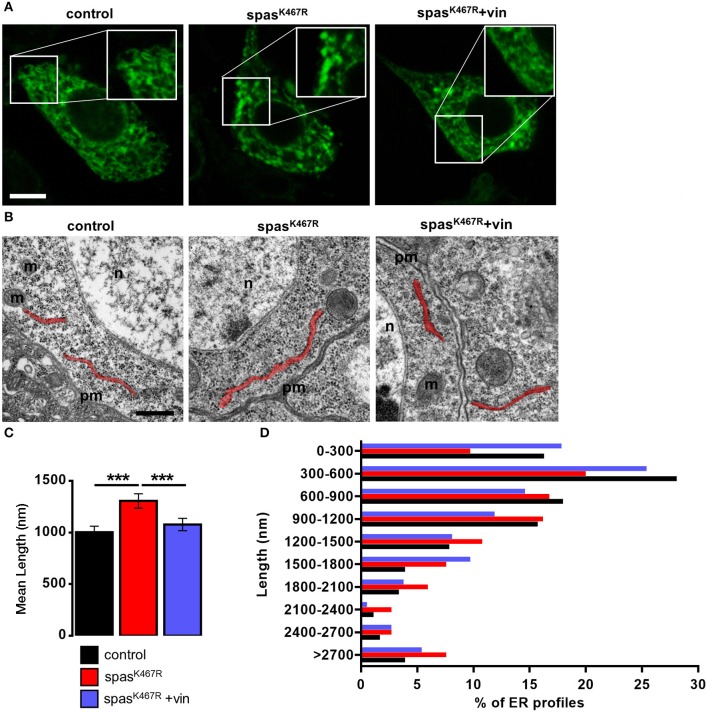
**(A)** Ventral ganglion motor neurons cell bodies of larvae of the indicated genotypes co-expressing BiP-sf-GFP-ER were imaged at the confocal microscope. Where indicated, spastin^K467R^ flies were raised in vinblastine-containing food (50 nM). Scale bar, 5 μm. **(B)** TEM images of ventral ganglion neuronal cell bodies of larvae expressing spastin^K467R^ and relative control; ER profiles are highlighted in red. pm, plasma membrane; n, nucleus; m, mitochondria. Scale bar, 500 nm. **(C)** Quantification of the mean length of ER profiles in TEM images for the indicated genotypes. Mean ± SEM, *n* ≥ 50 profiles. ***p < 0.001. **(D)** Distribution of ER profile length in TEM images. The percentage of measured profiles for each 300 nm-class is reported for the indicated genotypes.

Because MTs are known to regulate ER distribution and sheet/tubule balance (Terasaki et al., 1986; Lu et al., 2009), we examined ER morphology in fly neurons expressing spastin^K467R^. To visualize ER structure, we co-expressed the ER luminal marker BiP–sfGFP–HDEL (Summerville et al., 2016) under the control of the motoneuron-specific promoter D42-Gal4. In control motor neuron cell bodies, the ER appears mostly as a network of interconnected tubules (Figure 1A). In motor neurons expressing spastin^K467R^, ER morphology was markedly changed and extended fluorescent areas, likely representing ER sheets, were often present (Figure 1A, Supplementary Videos 1–3). Axons are believed to contain mainly tubular, smooth ER that tracks to axon termini (Tsukita and Ishikawa, 1976; Terasaki and Reese, 1994; Krijnse-Locker et al., 1995; Terasaki, 2018). Moving along the longest motor neurons from the cell body to the axon termini, the density of ER only slightly decreases in control larvae (Supplementary Figures 2A,B). In contrast, in larvae expressing spastin^K467R^, distal axons appear almost devoid of ER (Supplementary Figures 2A,B). This phenotype, although consistent with an impairment of axonal transport of ER tubules, could be also the result of a decrease in ER tubules amount. Noteworthy, western blotting analysis of BiP–sfGFP–HDEL amount in individuals expressing spastin^K467R^ or in controls indicated that the morphological alterations observed in cell bodies and axons does not affect total ER mass (Supplementary Figures 2C,D). To investigate in more depth the morphological change observed, we performed transmission electron microscopy (TEM) analysis of larval brains. The length of ER profiles measured in TEM thin sections, corresponding to a cut through sheet-like structures, reflects the organization of the ER (Puhka et al., 2012): a shift to longer profiles corresponds to an increase in sheets vs tubules ratio. The analysis of TEM thin sections revealed an increase in the length of ER profiles in spastin^K467R^-expressing neurons, compared to controls (Figures 1B,C). In particular, the relative abundance of profiles of the shortest classes (0–300 nm and 300–600 nm) is decreased in spastin^K467R^ expressing neurons, while an increase in profiles longer than 2 μm is evident (Figure 1D). This is consistent with an increase in ER sheets compared to tubules.

Altogether, these results suggest that the expression of a dominant-negative spastin mutant leads preferentially to the formation of ER sheets to the detriment of tubules.

### Neuronal Expression of Spastin^K467R^ Affects ER Ca^2+^ Handling

**Figure 2:**
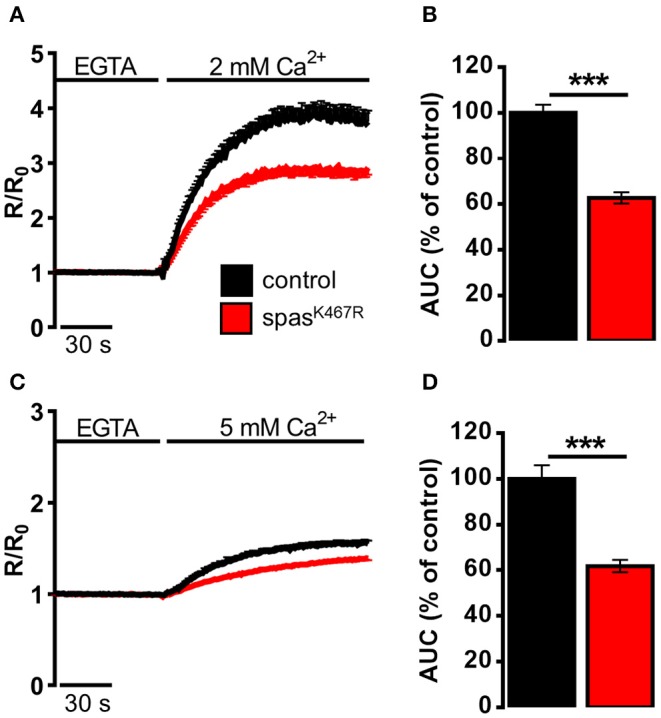
Ca^2+^ dynamics induced by **(A,B)** SOCE activation, **(C,D)** SOCE activation in K^+^-based medium (see Materials and Methods) in neurons dissociated from larval brains expressing spastin^K467R^, and relative controls, as detected by fura-2. **(A,C)** Cells were pre-treated with thapsigargin in a Ca^2+^-free medium for 8 min, to empty intracellular stores, and then **(A)** 2 mM or **(C)** 5 mM CaCl_2_ was added. In all panels, average traces are represented as R/R_0_ values of *n* > 100 cells. **(B,D)** Histograms report the average area under curve (AUC) values of the traces recorded. Data are presented as mean ± SEM of *n* >100 cells. ***p < 0.001.

We reasoned that such morphological changes would have an impact on definite ER functions that depend very much on the presence of tubular ER. Specifically, TAC-mediated movement of ER tubules is believed to be directly involved in SOCE activation, the process necessary to refill depleted ER Ca^2+^ stores. In order to investigate the impact of spastin^K467R^ expression on ER Ca^2+^ dynamics, and specifically on SOCE, neurons were isolated from larval brains expressing spastin^K467R^ under the control of a pan-neuronal promoter (elav-Gal4/UAS-spastin^K467R^), or from controls (elav-Gal4/+) (Supplementary Figure 3), loaded with the Ca^2+^ indicator fura-2 and examined by fluorescence microscopy. A typical protocol to elicit SOCE was applied to neurons: store depletion was induced by adding the SERCA inhibitor thapsigargin in a Ca^2+^-free medium; SOCE was then monitored upon CaCl_2_ addition (Ca^2+^, 2 mM). A large cytosolic Ca^2+^ concentration ([Ca^2+^]_c_) increase, followed by a sustained plateau, due to Ca^2+^ influx across the PM, was observed in control neurons (Figure 2A). The effect of spastin mutation on the Ca^2+^ influx activated by store depletion was quantified by calculating the area under the curve corresponding to the first 2 min of Ca^2+^ influx. A marked decrease in SOCE was observed in neurons from larvae expressing spastin^K467R^, compared to controls (37% reduction, *p* < 0.001; *n* = 350 control cells; *n* = 300 spastin^K467R^ cells; Figure 2B).

It is known that differences in PM potential alter the driving force for Ca^2+^ entry, thus potentially affecting the extent of SOCE (Penner et al., 1993). To nullify possible differences in membrane potential between the two genotypes, SOCE was measured as described above but in a medium where NaCl was iso-osmotically substituted by potassium-D-gluconate (K^+^-based medium, see Methods for details), causing the collapse of the membrane potential. A higher concentration of CaCl_2_ (5 mM) was applied, after emptying stores, to obtain an appreciable Ca^2+^ influx even under a reduced electrical gradient. Under such depolarizing conditions, the effect of spastin^K467R^ expression on SOCE was similar to that found in the standard Na^+^-containing medium (38% reduction, *p* < 0.001; *n* = 300 control cells; *n* = 280 spastin^K467R^ cells; Figures 2C,D). When basal SOCE was measured in the same cells, by simply adding back Ca^2+^ to cells bathed in a Ca^2+^-free medium, no difference was found between the two genotypes, neither in standard medium nor in K^+^-based medium (Supplementary Figures 4A–D).

**Figure 3:**
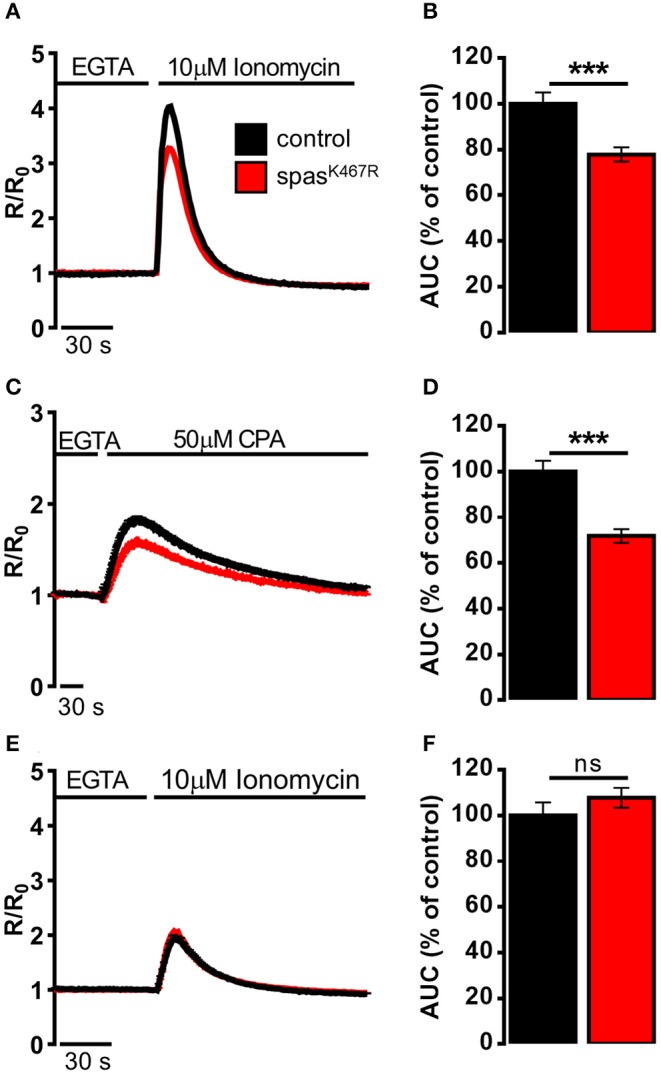
Ca^2+^ dynamics induced by **(A,B)** ionomycin, **(C,D)** CPA, **(E,F)** ionomycin after CPA-induced ER depletion in neurons dissociated from larval brains expressing spastin^K467R^, and relative controls, detected by fura-2. **(A,C,E)** Cells were stimulated with **(A,E)** 10 μM ionomycin or **(C)** 50 μM CPA in the presence of extracellular Ca^2+^ chelator EGTA. In all panels, average traces are represented as R/R_0_ values of *n* > 100 cells. **(B,D,F)** Histograms report the average area under curve (AUC) values of the traces recorded. Data are presented as mean ± SEM of *n* > 100 cells. ***p < 0.001.

The decrease in Ca^2+^ entry upon store depletion could cause a partial depletion of intracellular Ca^2+^ stores in spastin^K467R^ expressing neurons. To investigate this possibility, the Ca^2+^ ionophore ionomycin was applied to neurons bathed in a Ca^2+^-free medium containing the Ca^2+^ chelator EGTA. In this situation, the rise observed in [Ca^2+^]_c_ is due to the discharge of the majority of intracellular Ca^2+^ store pools (Figure 3A). The increase in [Ca^2+^]_c_ elicited by ionomycin was significantly reduced in spastin^K467R^ expressing neurons, relative to controls, as indicated by the area under the curve obtained upon ionomycin addition (22% reduction, *p* < 0.001; *n* = 90 control cells; *n* = 100 spastin^K467R^ cells; Figure 3B). A subsequent addition of monensin, in order to discharge any residual Ca^2+^ present in the acidic pool (Fasolato et al., 1991), did not result in an appreciable [Ca^2+^]_c_ increase in either genotypes (data not shown), indicating the relative low abundance of this type of Ca^2+^ stores in these cells. This result indicates that Ca^2+^ content of intracellular stores is diminished in cells expressing the spastin^K467R^ mutation. To determine whether the observed reduction was due to a specific partial depletion of the ER Ca^2+^ store, dissociated neurons were treated with the SERCA inhibitor CPA, thus inducing the passive release of Ca^2+^ from the organelle, resulting in a transient increase in [Ca^2+^]_c_ (Figure 3C). The amplitude of the increase in [Ca^2+^]_c_ reflects the Ca^2+^ content derived only from the ER and the cis/medial-Golgi, the main intracellular Ca^2+^ stores equipped with SERCA pumps (Lissandron et al., 2010; Wong et al., 2013). The increase in [Ca^2+^]_c_ elicited by CPA was significantly reduced in spastin^K467R^-expressing neurons, relative to controls (Figure 3C). The extent of such reduction, estimated calculating the area under the curve above resting [Ca^2+^]_c_ values, was 28% (*p* < 0.001; *n* = 220 control cells; *n* = 160 spastin^K467R^ cells; Figure 3D). Thus, ER Ca^2+^ content is diminished in cells expressing the spastin^K467R^ mutation. After CPA application, the discharge of residual Ca^2+^ pools, by ionomycin addition, did not show any differences between control and spastin^K467R^-expressing neurons (Figures 3E,F), indicating that the [Ca^2+^]_ER_ is primarily affected by spastin mutation. Of note, basal [Ca^2+^]_c_ content is not affected (Supplementary Figure 4E). Altogether, these data indicate that expression of spastin^K467R^ causes an impairment of the ER Ca^2+^ replenishment mechanism of SOCE. This likely results in a reduction of the steady-state [Ca^2+^]_ER_.

### Vinblastine Treatment Rescues ER Morphology and Ca^2+^ Handling Defects Induced by Spastin^K467R^ Expression

**Figure 4:**
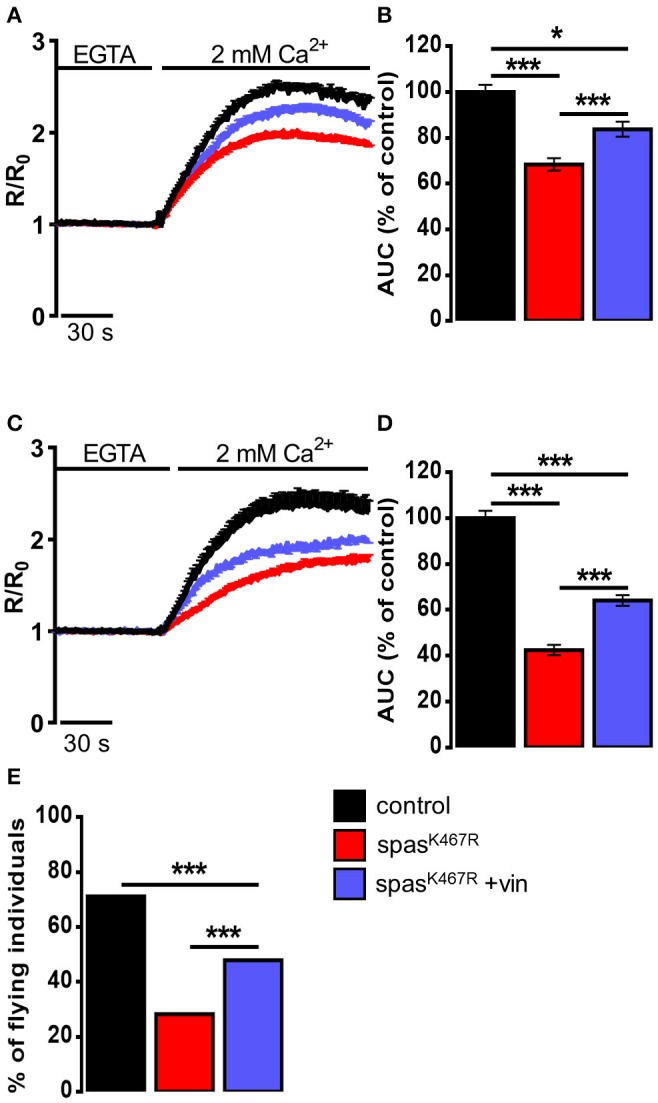
**(A–D)** Ca^2+^ dynamics induced by SOCE activation in neurons dissociated from larval brains expressing spastin^K467R^, and relative control, as detected by fura-2. Were indicated, vinblastine was applied: **(A,B)** during development, larvae were chronically treated with 50 nM vinblastine or **(C,D)** after dissociation, neurons were acutely treated with 1 μM vinblastine. Cells were pre-treated with thapsigargin for 8 min to empty intracellular stores, then 2 mM CaCl_2_ was added. Traces are presented as average R/R_0_ values of *n* > 100 cells. **(B,D)** Histograms reporting the average area under curve (AUC) values of the corresponding traces. Data are presented as mean ± SEM of *n* > 100 cells. *p < 0.05; ***p < 0.001. **(E)** Flight ability, assayed by the cylinder drop test, of animals expressing spastin^K467R^ and relative control. Where indicated, animals were chronically exposed to vinblastine (50 nM). Data are presented as the percentage of flying individuals. Mean of *n* > 100 cells. ***p < 0.001.

In order to assess whether MT cytoskeleton impairment was directly responsible for ER morphology and Ca^2+^ handling defects observed in spastin^K467R^-expressing flies, and to exclude other possible effects of mutant spastin expression, we exploited a pharmacological approach. It has been shown that administration of low concentrations of the MT-targeting drug vinblastine rescued the excessive stabilization of MTs in spastin^K467R^-expressing flies (Orso et al., 2005). We thus administered vinblastine to control and spastin^K467R^ flies by adding the drug to the food at a concentration of 50 nM (Orso et al., 2005). When we examined the fluorescence of the ER marker BiP–sfGFP–HDEL (Summerville et al., 2016), we found that exposure to the MT-targeting drug resulted in recovery of ER morphology in flies expressing spastin^K467R^ (Figure 1A). The rescue is confirmed also by the quantification of ER profiles length in TEM thin sections (Figures 1B,C). Moreover, in neurons dissociated from brains of the same larvae, we evaluated SOCE, as described above. We found that the reduction in the Ca^2+^ entry following stores depletion, observed in neurons derived from larvae expressing spastin^K467R^, was partially recovered by vinblastine treatment (Figures 4A,B), indicating that the drug-induced destabilization of hyper-stabilized MTs rescues the spastin^K467R^-induced SOCE defects.

It has been shown that loss of MT polymers in response to vinblastine occurs very rapidly, starting in as little as 30 min (Harkcom et al., 2014). In order to further demonstrate that the rescue of MT cytoskeleton is directly responsible for the recovery of SOCE impairment observed in spastin^K467R^-expressing flies, we acutely applied vinblastine (1 μM) on neurons dissociated from spastin^K467R^ larvae raised in the absence of drug in the food. Application of vinblastine for 40 min before SOCE activation and visualization was able to induce a partial rescue in the extent of Ca^2+^ entry, compared to untreated spastin^K467R^-expressing neurons (Figures 4C,D). This result indicates that reestablishment of proper MT organization is sufficient to rescue the process of Ca^2+^ entry upon stores depletion, affected by the spastin mutant.

Pan-neural downregulation of *dStim* or *dOrai* leads to a significant reduction of SOCE and ER [Ca^2+^] in primary neuronal cultures (Venkiteswaran and Hasan, 2009). Reduced SOCE has been shown to affect fly neuronal functions, in particular a significant loss of flight has been observed. In order to assess whether the defect in SOCE observed upon spastin^K467R^ expression was also associated with an impairment in flight, we performed the “cylinder drop” test assay, which revealed a defect in the flight ability of spastin^K467R^-expressing flies (42% reduction of spastin^K467R^ compared to control, *p* < 0.001; *n* = 100 control cells; *n* = 100 spastin^K467R^ cells; Figure 4E). This defect was partially rescued when flies were raised in vinblastine-containing food (Figure 4E).

## Discussion

Ca^2+^ signals regulate fundamental aspects of neuronal function and physiology and contribute in determining the morphology of neural circuits (Berridge, 1998; Borodinsky and Spitzer, 2007). Traditionally, most of these signals were attributed to the entry of Ca^2+^ from the extracellular *milieu* through voltage-operated channels or ionotropic receptors. However, the “Ca^2+^ toolkit” components related to Ca^2+^ release from intracellular stores are also present in neurons. Increasing evidence suggests that also neurons rely on SOCE and its dysregulation may participate in the pathogenesis of diverse neurodegenerative diseases, such as Alzheimer's, Parkinson's, Charcot-Marie-Tooth (Secondo et al., 2018).

The aim of this study was to determine the influence of neuronal MT cytoskeleton architecture on the process of SOCE, the opening of PM Ca^2+^ channels that follows the release of Ca^2+^ from intracellular stores. The molecular mechanism of cytoskeleton regulation over the relocation of STIM to ER-PM junctions during SOCE is not fully understood. It appears clear that coordinated interplay between different molecules is necessary to mediate the transient formation of ER-PM junctions (Grigoriev et al., 2008; Sharma et al., 2013; Maléth et al., 2014; Woo et al., 2016), but whether the integrity of cytoskeleton is needed for proper SOCE activation is not clear. Opposing data are present in literature, suggesting both inhibition (Oka et al., 2005; Smyth et al., 2007) or potentiation (Galán et al., 2011) of SOCE in the presence of MT-depolymerizing agents (Russa et al., 2009; Martin-Romero et al., 2017). To sort this out, we performed an *in vivo* approach in *Drosophila melanogaster* exploiting an endogenous means to manipulate MT dynamic instability. We expressed in Drosophila a variant of the MT-severing protein spastin carrying an amino acid substitution known to function as a dominant-negative, thus inducing MT hyper-stabilization. Our results clearly indicate that the process of Ca^2+^ entry upon ER Ca^2+^ depletion is negatively affected by MT stabilization. This impairment results in a reduced ER Ca^2+^ content without, however, affecting cytosolic basal Ca^2+^ levels.

The phenotypes we observed upon neuronal expression of spastin^K467R^ recapitulate those observed upon dStim or dOrai reduction in flies. Pan-neural downregulation of *dStim* or *dOrai* leads to a significant decrease in SOCE and ER [Ca^2+^] in primary larval neurons (Venkiteswaran and Hasan, 2009). Reduced SOCE has been shown to affect fly neuronal functions, in particular a significant loss of flight has been observed, accompanied by the loss of rhythmic flight patterns (Venkiteswaran and Hasan, 2009), indicating that, in neurons, the replenishment of intracellular Ca^2+^ stores is required for Drosophila flight. We observed a similar defect in flight ability, tested in the cylinder drop assay, in flies expressing spastin^K467R^. This phenotype is specific, since dSERCA mutant flies, where stored Ca^2+^ is decreased but SOCE is increased, do not display flight defects (Banerjee et al., 2006; Venkiteswaran and Hasan, 2009).

We found that spastin^K467R^-induced MT stabilization causes a change in ER morphology, shifting ER sheets/tubules balance toward the formation of sheets. Interestingly, an accumulation of ER sheets has also been reported in mammalian cells upon treatment with MT-depolymerizing (Terasaki et al., 1986; Joensuu et al., 2014) as well as -stabilizing agents (Joensuu and Jokitalo, 2015), suggesting that ER morphology is similarly affected upon MT cytoskeleton disruption or hyper-stabilization.

It is widely assumed that the structural heterogeneity of the ER contributes to its functional compartmentalization. Despite the fact that a clear-cut attribution of function to either sheets or tubules has yet to be defined, tubules appear to perform some specific functions. The involvement of TAC-mediated movement on MT tips suggests that the regulated process of SOCE is allocated specifically to ER tubules. The data we obtained allow us to speculate that MT disorganization and SOCE impairment are causally linked by the alteration of ER morphology observed upon spastin^K467R^ expression in flies: MT hyperstabilization shifts the ER sheets/tubules balance in favor of ER sheets; this in turn affects SOCE, a specific ER function that relies on the physical movement of ER tubules toward the PM. However, MT dynamics itself is critical to generate pushing and pulling forces during polymerization and depolymerization, respectively (Inoué and Salmon, 1995) providing the force required for membrane movement that can result in membrane translocation from one point to another within the cell. For this reason, we cannot exclude that MT impairment *per se* is responsible for the observed reduction of SOCE.

Beneficial effects from treatments with vinblastine have been reported in flies expressing spastin^K467R^ (Orso et al., 2005). By recovering MT organization, vinblastine rescues NMJs morphology and function, together with fly viability and climbing defects (Orso et al., 2005). We demonstrate that vinblastine treatment is able to rescue also ER morphology, Ca^2+^ handling defects and flight ability, indicating that MT impairment is the earliest responsible for all the phenotypes observed. Notably, acute vinblastine treatment (40 min application on dissociated neurons) is sufficient to rescue SOCE and ER Ca^2+^ content. Within this time-window, transcriptional activation is unlikely to occur, suggesting that the level of Ca^2+^ handling proteins, and in particular of the SOCE machinery, is expected to be unaltered and the defects observed are directly ascribable to a MT dynamics impairment.

Tubulin represents about 4% of total cellular proteins in many cultured cells, however it reaches 25% in the brain (Zhai and Borisy, 1994). In axons and dendrites, MTs serve as the major railways for organelles and other cargoes and dysfunctional MT scaffolding has been primarily associated with impaired transport. Neuronal functionalities, including learning and memory, are associated with the normal functioning of dendritic spines that could be compromised if organelles and proteins do not reach their proper location. In addition to this evident relationship between MT organization and neuronal function, our present work supports the idea that other fundamental cellular mechanism, namely ER shape and function, are affected by MT disorganization caused by mutant spastin expression. This is critically important considering that increasing evidence suggests the presence of a causative link between derangement of ER morphology/function and the pathogenesis of HSPs.

## Data Availability Statement

The datasets generated for this study are available on request to the corresponding author.

## Author Contributions

DP conceived the work. NV and RN performed the experiments. NV and NR analyzed the results. PP and AD contributed to the interpretation of the results. DP wrote the manuscript. DP and PP secured funding. All the authors revised the manuscript.

### Conflict of Interest

The authors declare that the research was conducted in the absence of any commercial or financial relationships that could be construed as a potential conflict of interest.
