# Supplementary material for: Microtubules Stabilization by Mutant Spastin Affects ER Morphology and Ca2+ Handling
Source: Front Physiol. 2019 Dec 20;10:1544. doi: 10.3389/fphys.2019.01544 (PMC6933510; doi:10.3389/fphys.2019.01544)
Supplement: Supplementary file 1 [file Data_Sheet_1.pdf]

Supplementary Material

1 Supplementary Figures

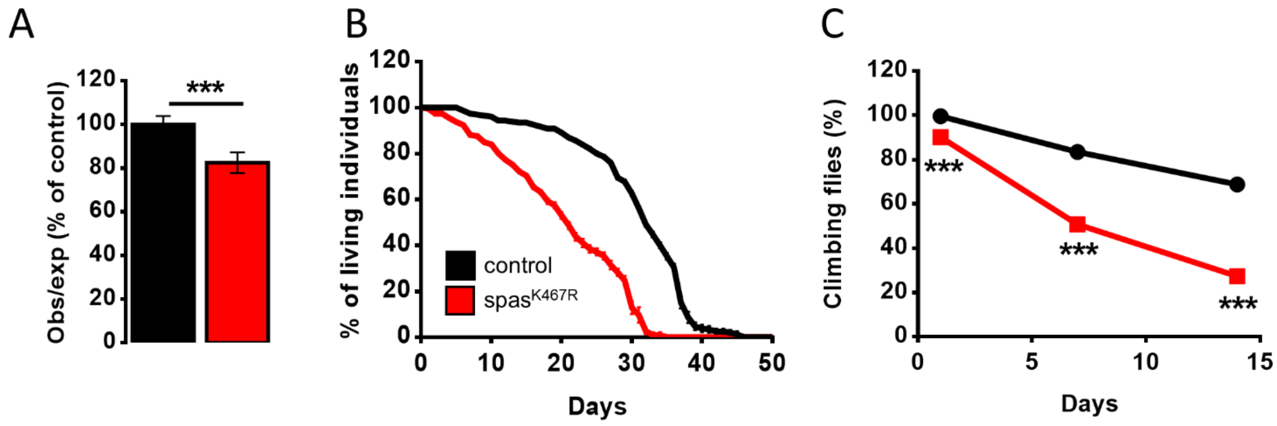

Supplementary Figure 1. (A) Flies of the two genotypes (Control, black; red, spastin^{K467R}) were counted at the eclosure to define their birth rate. Data are presented as observed/expected flies ratio, mean ± SEM of n > 2000 flies, *** $p < 0.001$. **(B)** Evaluation of fly survival: flies were separated in tubes (20 individuals/each) and counted every 2 days through ageing. Data represented as percentage of living flies over 40 days. n > 200 flies. **(C)** Evaluation of fly climbing ability: flies were tapped at the bottom of a graduated tube. Flies able to cross a 10 cm mark in 60 s were counted as climbing flies. Data shown as percentage of flies able to cross the line at day 1, 7 and 14. n > 200 flies.

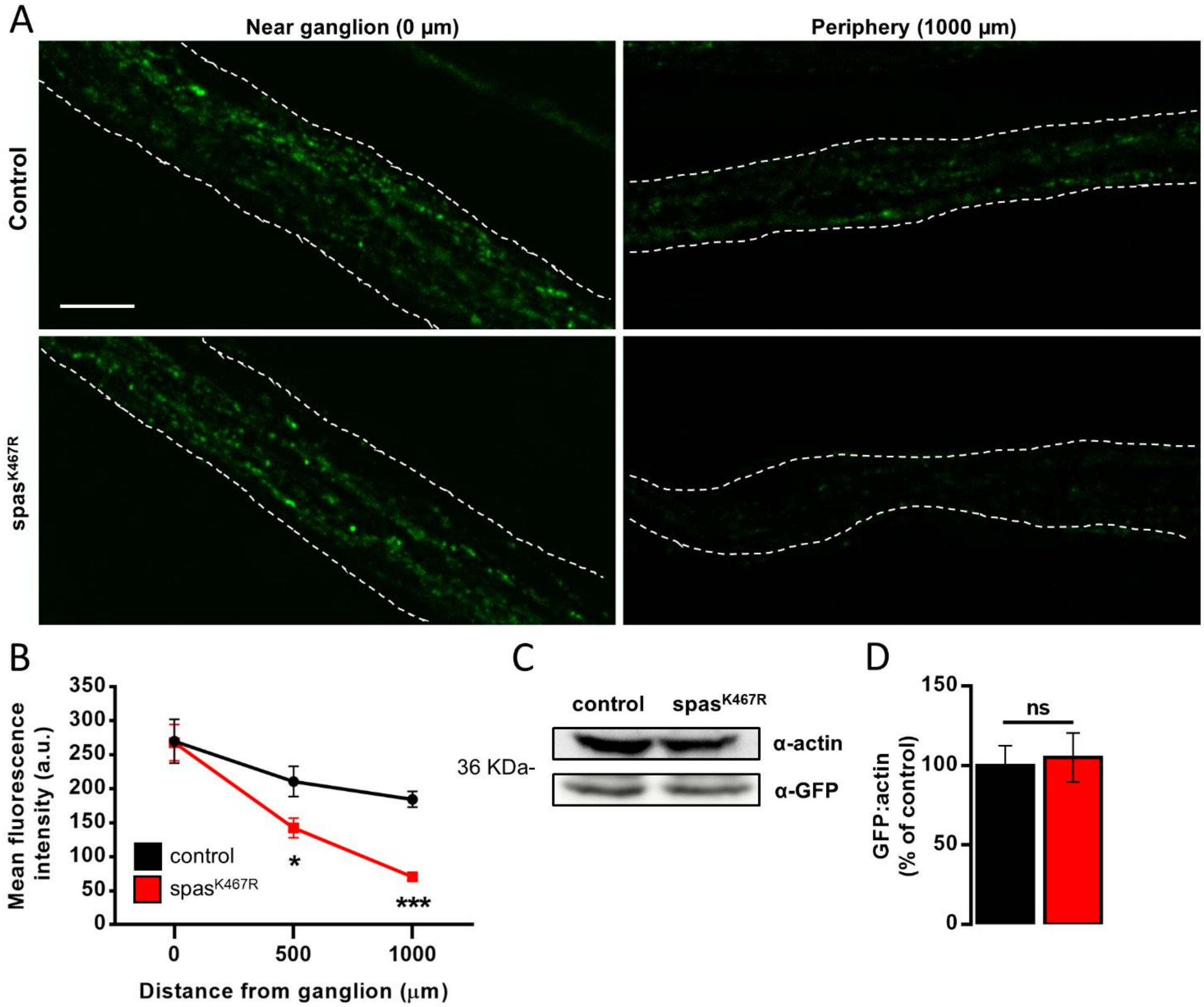

Supplementary Figure 2. (A) Confocal images of motor neurons nerves of larvae of the indicated genotypes co-expressing BiP-sf-GFP-ER. Pictures on the left were acquired at the level of A2 larval segment, *i.e.*, near cell bodies, while pictures on the right were acquired at the level of larval segment A5, *i.e.*, far from the cell bodies. Scale bar, 50 µm. **(B)** Quantification of BiP-sf-GFP-ER mean fluorescence intensity in regions located near the ganglion (0 nm), along the axon (500 nm) and at the end of the larval body (1000 nm). Data are presented as mean ± SEM of $n > 7$ axons. * $p < 0.05$, *** $p < 0.001$. **(C,D)** Representative western blot **(C)** and quantification, normalized to β-actin levels **(D)**, of BiP-sf-GFP-ER amount in motor neurons of individuals expressing spastin^{K467R}, compared to controls. Mean ± SEM, n = 3.

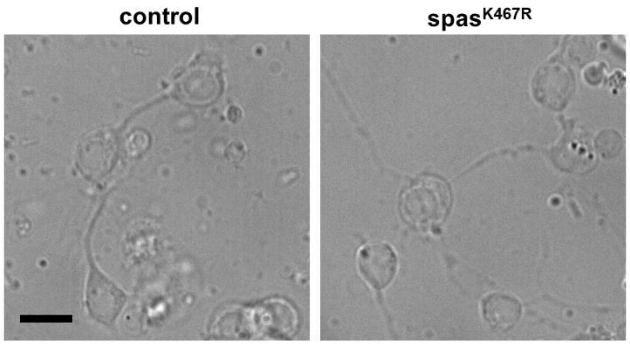

Supplementary Figure 3. Bright-field images of neurons isolated from brains of larvae expressing spastin^{K467R} and controls. Images have been taken 16 hours after plating, before starting Ca^{2+} imaging experiments. Scale bar 10 µm.

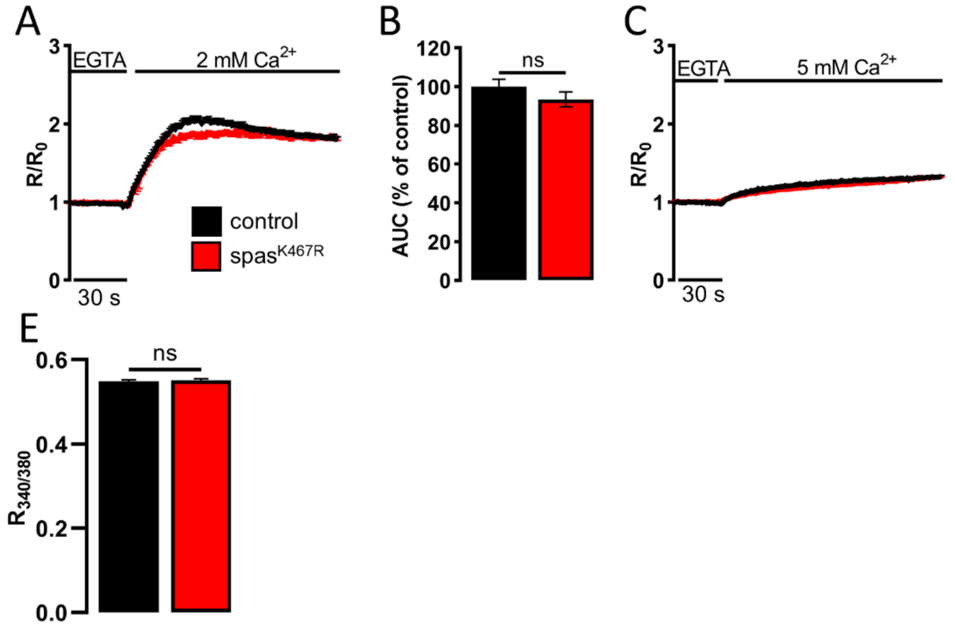

Supplementary Figure 4. Ca^{2+} dynamics induced by basal SOCE activation in **(A,B)** standard M1 medium or **(C,D)** K^{+}-based medium (see Materials and Methods) in neurons dissociated from larval brains expressing spastin^{K467R}, and relative controls, as detected by fura-2. Cells were kept in Ca^{2+}-free, EGTA-containing medium for 30 s and then **(A)** 2 mM or **(C)** 5 mM $CaCl_2$ was added. Traces are presented as average R/R_0 values of n > 200 cells. **(B,D)** Histograms reporting the average area under curve (AUC) values of the traces. **(E)** Basal [Ca^{2+}] level measured in neurons bathed in standard M1 medium. Basal ratio values are shown. Data are presented as mean ± SEM of n > 200 cells.

2 Supplementary Videos

Supplementary Video 1. Z-stack of ventral ganglion motor neuron cell bodies of a control larva expressing ER marker BiP-sf-GFP-ER. Scale bar 5 µm.

Supplementary Video 2. Z-stack of ventral ganglion motor neuron cell bodies of a larva co-expressing spastin^{K467R} and ER marker BiP-sf-GFP-ER. Scale bar 5 µm.

Supplementary Video 3. Z-stack of ventral ganglion motor neuron cell bodies of a larva co-expressing spastin^{K467R} and ER marker BiP-sf-GFP-ER, raised in vinblastine-containing food (50 nM). Scale bar 5 µm.
